# Needs Assessment in Care of Adults With Anorectal Malformations and Exstrophy-Epispadias Complex in Germany

**DOI:** 10.3389/fped.2018.00392

**Published:** 2018-12-19

**Authors:** Anne Karoline Ebert, Nadine Zwink, Nicole Schwarzer, Lilian Brunner, Heiko Reutter, Ekkehart Jenetzky, Johannes Huber, Barbara Ludwikowski

**Affiliations:** ^1^Department of Pediatric Urology, University Hospital for Urology and Pediatric Urology Ulm, Ulm, Germany; ^2^Department of Pediatric and Adolescent Psychiatry and Psychotherapy, University Medical Centre, Johannes Gutenberg University Mainz, Mainz, Germany; ^3^SoMA e.V., Self-Help Organisation for People With Anorectal Malformation, Munich, Germany; ^4^Self-Help Organisation for People With Bladder exstrophy-epispadias complex, Freital, Germany; ^5^Institute of Human Genetics, University of Bonn, Bonn, Germany; ^6^Department of Neonatology and Pediatric Intensive Care, Children's Hospital, University of Bonn, Bonn, Germany; ^7^Child Center Maulbronn GmbH, Hospital for Pediatric Neurology and Social Pediatrics, Maulbronn, Germany; ^8^University Hospital for Urology, University Medical Center Carl Gustav Carus, Dresden, Germany; ^9^Department of Pediatric Surgery and Pediatric Urology, Medical Center for Children and Adolescents AUF DER BULT, Hannover, Germany

**Keywords:** anorectal malformation (ARM), exstrophy-epispadias complex (EEC), health care, long-term quality of life issues, medical aftercare

## Abstract

**Introduction:** Medical needs of adults with anorectal malformations (ARM) and the exstrophy-epispadias complex (EEC) are not fully understood. Therefore, the aim of the study was to evaluate how affected individuals get along with the current national medical care and what their medical or social long-term requirements are.

**Patients and Methods:** Between 11/2014-07/2016 all adult members (≥18 years, ARM *n* = 113, EEC *n* = 126) of the German self-help organizations SoMA e.V. and Blasenekstrophie/Epispadie e.V. were contacted via email or post and asked to fill out an anonymous online questionnaire regarding medical requirements, treatment satisfaction, daily life impairment and expectations regarding physicians soft skills. The results were compared between both groups and male and female participants.

**Results:** 56 participants with ARM (median age 26 years, IQR 19-38) and 52 participants with EEC (median age 31 years, IQR 22-37) filled in the questionnaire completely. Forty-five percent of the ARM and 67% of the EEC participants contacted an urologist. A general surgeon was visited by 23% of the ARM individuals, a peadiatric surgeon by 20%. Although 60% of the females with ARM and 82% of the females with EEC assessed gynecological counseling as helpful or neutral, a small subgroup of ARM females (*n* = 6, 16%; 70% non-isolated ARM or ARM with Hirschsprung disease and additional associated anomalies) were not satisfied. The majority of both groups reported no or only minor daily life impairment (*p* = 0.38). Professional knowledge, paying attention to patients' concerns, having empathy and taking enough time was important for over 90% of all participants. Thirty-eight percent of the ARM and 27% of the EEC individuals needed psychological support. Most medical consultations were judged to be helpful.

**Conclusion:** Although adults with ARM and EEC being a self-help organization member and thus well informed and generally cope well, participants expressed their wish for expert counseling regarding family planning, reconstructive procedures, continence management, urological care and social welfare issues. Furthermore, specific expert consultations for gynecological issues in a subgroup of ARM females, mainly non-isolated, might be required. Actual needs of adults with rare conditions must be better clarified to improve medical care beyond childhood and adolescence.

## Introduction

There is no doubt that long-term medical care is crucial for individuals with rare congenital anomalies, such as anorectal malformations (ARM) and exstrophy-epispadias complex (EEC). There is growing evidence that long-term care does not only improve satisfaction and quality of life (QoL) of the affected individuals, but also reduces complications and therefore morbidity (1–3). From physicians' perspective there is an increasing knowledge in literature reviews about “what to expect in adult life” for urological congenital conditions such as posterior urethral valves, EEC, hypospadias, spina bifida and Klinefelter‘s syndrome with a primary focus on long-term complications or their prevention (4–9). Additionally, the American Urological Associatian (AUA) developed helpful consensus recommendations for clinical important index situations, such as pregnancy in EEC or in reflux nephropathy (7). Further clinical research focusing on “worries and needs” of the affected patients was rarely performed (10). Although, during recent years first experiences with urological transition programs were published, transition programs are implemented only in a few countries worldwide (11). From physicians and physicians societies perspective and in the view of health care policy there is still an ongoing debate about which physician should take care for patients with congenital conditions and how these physicians should be trained for providing adequate support during and after transition (12, 13).

The aim of this study was to explore the use of currently available medical support in adults with ARM and EEC in Germany and their current medical issues, worries and needs. Therefore, we evaluated the access to medical professionals and the need for counseling relating to medical and social issues.

## Materials and Methods

### Study Population

Members of the two German self-help organizations SoMA e.V. (www.soma-ev.de; actually more than 300 affected members) and Blasenekstrophie/Epispadie e.V. (www.blasenekstrophie.de; actually more than 300 affected members) at least 18 years old were identified and contacted by email or post. Individuals who expressed the wish to take part got a link to a complete anonymous online questionnaire. For those who did not like to fill in an online questionnaire, a paper-based anonymous questionnaire was offered and sent by post. After 6 weeks, 3 and 6 months a reminder letter was sent by email or post. In addition, the two self-help organizations informed their members at their annual meetings and asked them to attend the study.

This online-questionnaire was completely anonymous and no patient identifying data were collected, so the participants could not be re-identified or re-contacted for more detailed informations. Therefore, written informed consent and an ethics statement were not required. Only completely answered questionnaires were included for analysis.

The questionnaire [Data Sheet 1 (Final questionnaire translated_EEC) + Data Sheet 2 (Final questionnaire translated_ARM)] was designed by the authors, among them the board leaders of the self-help organizations, and constructed by the open source survey application LimeSurvey®. We translated the original German questionnaire in English. Before use, a pre-test was carried out. Three adult members of the self-help organizations, not working in the medical or scientific field and not involved in the design process, answered the questionnaire independently of each other. They gave a positive feedback about the understandability, the questions' relevance and the necessary time frame to fill out the questionnaire. Basic epidemiological data referred exclusively to gender, age and phenotype. For a standardized phenotype description the EEC classification by Gearhart & Jeffs was used (14). ARM phenotype without additional anomalies was classified as isolated ARM, ARM with additional anomalies was defined as non-isolated ARM (15). Additionally, ARM with Hirschsprung disease (HSCR) was categorized separately. All questions were constructed in the same pattern: “Please indicate which professional help or medical treatment you used during the last 24 months. Please state how useful this consultation or treatment was for you.” As the aim of the study was to explore the medical support in everyday life, participants got the advice not to refer to medical specialists they might have met at self-help group meetings. Answers were categorized into “no contact,” “the contact or treatment was helpful,” “the contact or treatment was neutral” or “the contact or treatment was not helpful.” Additionally, there was enough space at the end of each question for an open free-text comment. Furthermore, participants were asked which professional soft skills their treating physicians' should have, such as medical expertise, paying attention to patients' concerns, having empathy, taking enough time, collaboration with other disciplines or the self-help groups as well as having been treating patients with ARM/EEC before [Data Sheet 1 (Final questionnaire translated_EEC) + Data Sheet 2 (Final questionnaire translated_ARM)]. The importance should the participants mark on a Lickert scale from 0 = not important to 5 = very important. Finally, the participants were asked to indicate the degree of daily life impairment caused by the anomaly, ranging from 0 = no impairment to 10 = severe impairment.

### Statistical Analysis

Descriptive data of both study populations are presented in absolute and relative frequencies. To assess possible differences between the two groups ARM/EEC as well as female and male participants Fisher's exact test was used, for possible differences in age distribution between ARM and EEC groups the Wilcoxon test. Statistical significance was defined by *p* < 0.05. Analyses were performed by the statistics software SAS©, version 9.4 (SAS Institute Inc., Cary, N.C., United States).

## Results

Eighty-four individuals with ARM and 72 individuals with EEC participated in this study. The overall response rate of the contacted adult self-help group members was 35% in the ARM and 75% in the EEC cohort (*p* < 0.0001). Incomplete questionnaires were excluded [28 (33%) in the ARM, 20 (28%) in the EEC group]. Finally, data of 56 (67%) participants with ARM and 52 (72%) participants with EEC were analyzed (*p* = 0.49). No contact to the self-help groups during the past 2 years were documented in 18 (32%) ARM and 28 (54%) EEC adults (*p* = 0.03). Epidemiological data can be found in Table 1. There was a significant difference regarding to male-to-female ratio between the participants with ARM and EEC (*p* = 0.0005). With reference to age ranges, no significant difference was found (*p* = 0.32). Phenotype distribution of the ARM participants showed 30% isolated ARM and 63% non-isolated ARM. In EEC the predominance of the CBE phenotype was documented (85%).

**Table 1:** Epidemiological data of the participants.

	**ARM (*n* = 56)**	**EEC (*n* = 52)**
**GENDER**		
Female	38 (68%)	17 (33%)
Male	18 (32%)	34 (65%)
Other (DSD)	0	1 (2%)
No answer	0	0
**AGE (IN YEARS)**		
Median	26	31
IQR	19–38	22–37
Min, Max	18, 62	18, 58
Phenotype		
**ARM**		
Isolated ARM	17 (30%)	
Non-isolated ARM	35 (63%)	
ARM with Hirschsprung disease	1 (2%)	
ARM without Hirschsprung disease	52 (93%)	
EEC		44 (85%)
Classical bladder exstrophy (CBE)		4 (8%)
Epispadias (E) I		0
Epispadias (E) II		1 (2%)
Epispadias (E) III		1 (2%)
Epispadias, unknown grading		0
Cloacal exstrophy (CE)	0	1 (2%)
Other, not yet specified	3 (5%)	1 (2%)
No answer/missing phenotype		

Medical and paramedical consultations and their usefulness observed by the participants are shown in Tables 2, 3. Thirty-five adults with EEC contacted an urologist, 23 of them (44%) marking the contact helpful, 11 (21%) neutral and one (2%) not helpful. Further 18 (35%) marked to have a helpful contact to a pediatric urologist, neutral contact had four (8%). Seventeen (30%) adults with ARM were satisfied with an urological consultation, six (11%) were neutral and two (4%) reported their urological consultation not to be helpful. In general, the item “contact or treatment not helpful” was marked in most subspecialities in a minority of 2–4%. Although 47% of females either with ARM (*n* = 18) or EEC (*n* = 8) stated that their gynecological contact or treatment was helpful, and further five females with ARM (13%) and six females with EEC (35%) determined their gynecological consultation as neutral, a small subgroup of six ARM females (16%) were not satisfied. One of these females had an isolated ARM, four females a non-isolated ARM with additional anomalies (67%) and one further female had ARM with Hirschsprung disease and additional malformations (17%).

**Table 2:** Medical consultations and their usefulness observed by the participants.

	**No contact**	**Contact or treatment helpful**	**Contact or treatment neutral**	**Contact or treatment not helpful**	**No answer**	***P*-value**
Pediatric surgeon						*p* = 0.74
ARM	37 (66%)	9 (16%)	2 (4%)	0	8 (14%)	
EEC	34 (65%)	6 (12%)	1 (2%)	0	11 (21%)	
Surgeon						*p* = 0.29
ARM	36 (64%)	8 (14%)	3 (5%)	2 (4%)	7 (13%)	
EEC	36 (69%)	6 (12%)	0	0	10 (19%)	
Urologist						
ARM	25 (45%)	17 (30%)	6 (11%)	2 (4%)	6 (11%)	
EEC	11 (21%)	23 (44%)	11 (21%)	1 (2%)	6 (12%)	
Pediatric urologist						–
ARM	-	-	-	-	-	
EEC	24 (46%)	18 (35%)	4 (8%)	0	6 (12%)	
Proctologist						*p* = 0.46
ARM	40 (71%)	6 (11%)	3 (5%)	1 (2%)	6 (11%)	
EEC	36 (69%)	3 (6%)	2 (4%)	0	11 (21%)	
Gynecologist^*^						*p* = 0.23
ARM (n = 38)	5 (13%)	18 (47%)	5 (13%)	6 (16%)	4 (11%)	
EEC (n = 17)	2 (12%)	8 (47%)	6 (35%)	0	1 (6%)	
Psychologist						*p* = 0.099
ARM	28 (50%)	20 (36%)	1 (2%)	0	7 (13%)	
EEC	28 (54%)	9 (17%)	3 (6%)	2 (4%)	10 (19%)	
Nephrologist						*p* = 0.70
ARM	41 (73%)	5 (9%)	3 (5%)	0	7 (13%)	
EEC	34 (65%)	5 (10%)	2 (4%)	0	11 (21%)	
Gastroenterologist						*p* = 0.52
ARM	38 (68%)	3 (5%)	4 (7%)	2 (4%)	9 (16%)	
EEC	39 (75%)	2 (4%)	1 (2%)	0	10 (19%)	
General practicioner						*p* = 0.31
ARM	8 (14%)	21 (38%)	22 (39%)	1 (2%)	4 (7%)	
EEC	8 (15%)	27 (52%)	11 (21%)	1 (2%)	5 (10%)	

* *only female participants*.

**Table 3:** Paramedical consultations and their usefulness observed by the participants.

	**No contact**	**Contact or treatment helpful**	**Contact or treatment neutral**	**Contact or treatment not helpful**	**No answer**	***P*-value**
Paramedical						*p =* 0.085
conservative continence management						
ARM	42 (75%)	5 (9%)	2 (4%)	0	7 (13%)	
EEC	38 (73%)	0	2 (4%)	0	12 (23%)	
Osteopathy						*p =* 0.11
ARM	35 (63%)	13 (23%)	0	0	8 (14%)	
EEC	33 (63%)	5 (10%)	2 (4%)	1 (2%)	11 (21%)	
Nutritional consulting						*p =* 0.16
ARM	44 (79%)	0	3 (5%)	0	9 (16%)	
EEC	38 (73%)	2 (4%)	0	1 (2%)	11 (21%)	
Physiotherapy						*p =* 0.099
ARM	28 (50%)	20 (36%)	1 (2%)	0	7 (13%)	
EEC	28 (54%)	9 (17%)	3 (6%)	2 (4%)	10 (19%)	
Alternative practitioner						*p =* 0.70
ARM	36 (64%)	9 (16%)	0	1 (2%)	10 (18%)	
EEC	32 (62%)	8 (15%)	2 (4%)	0	10 (19%)	
Community health and social workers						*p =* 0.75
ARM	41 (73%)	3 (5%)	1 (2%)	0	11 (20%)	
EEC	40 (77%)	1 (2%)	0	0	11 (21%)	
Self-help group						*p* = 0.082
ARM	18 (32%)	24 (43%)	5 (9%)	0	9 (16%)	
EEC	28 (54%)	15 (29%)	1 (2%)	0	8 (15%)	

**Figure 1:**
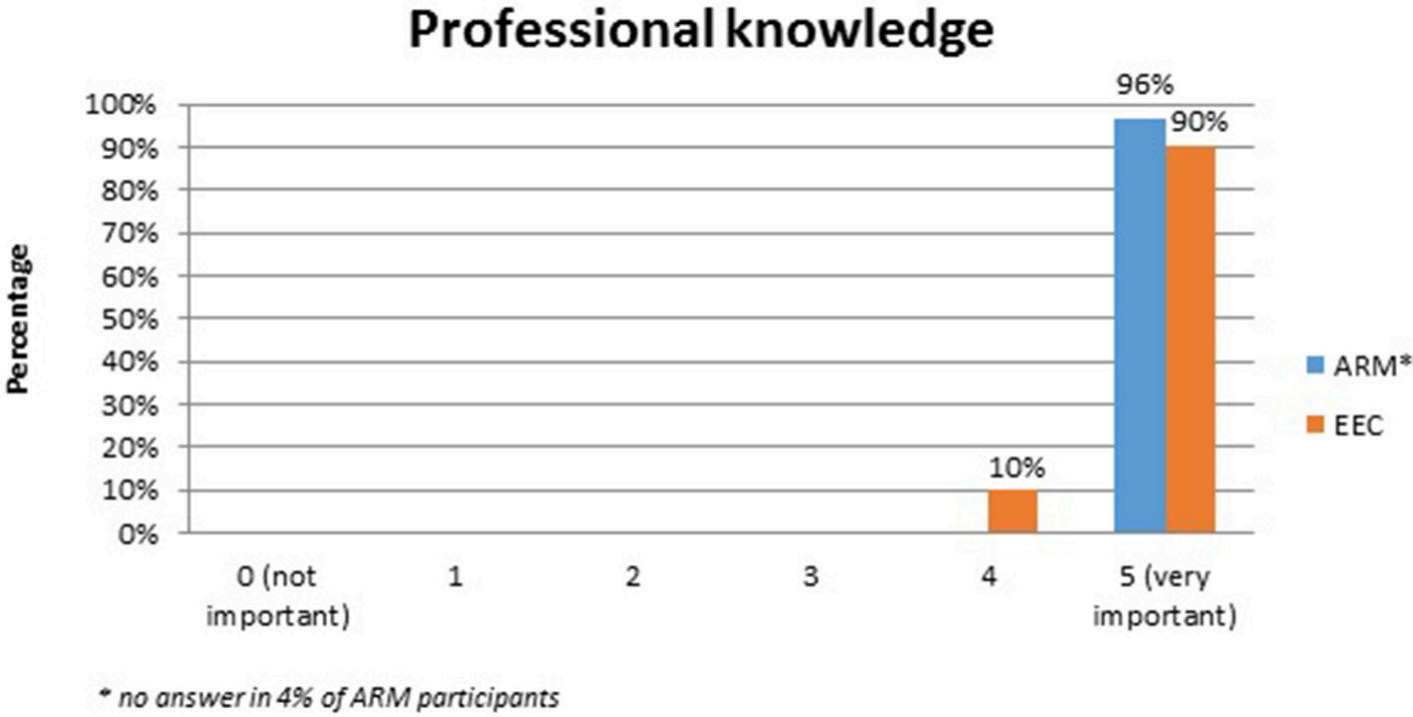
Expectations of adults with ARM and EEC regarding the soft skills of the treating physician: professional knowledge.

**Figure 2:**
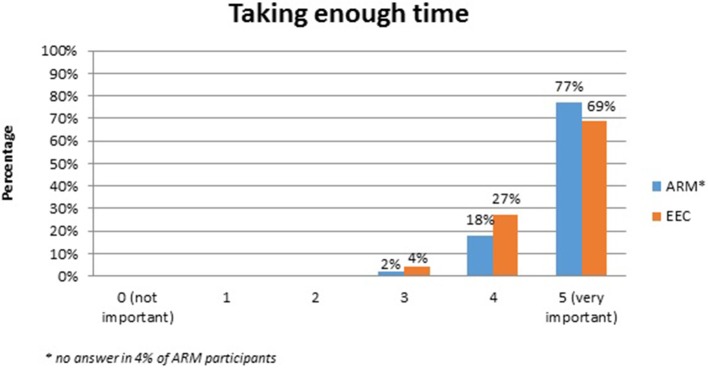
Expectations of adults with ARM and EEC regarding the soft skills of the treating physician: taking enough time.

**Figure 3:**
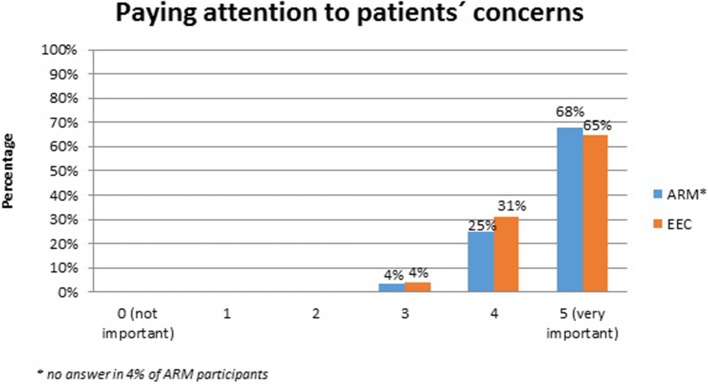
Expectations of adults with ARM and EEC regarding the soft skills of the treating physician: paying attention to patients concerns.

**Figure 4:**
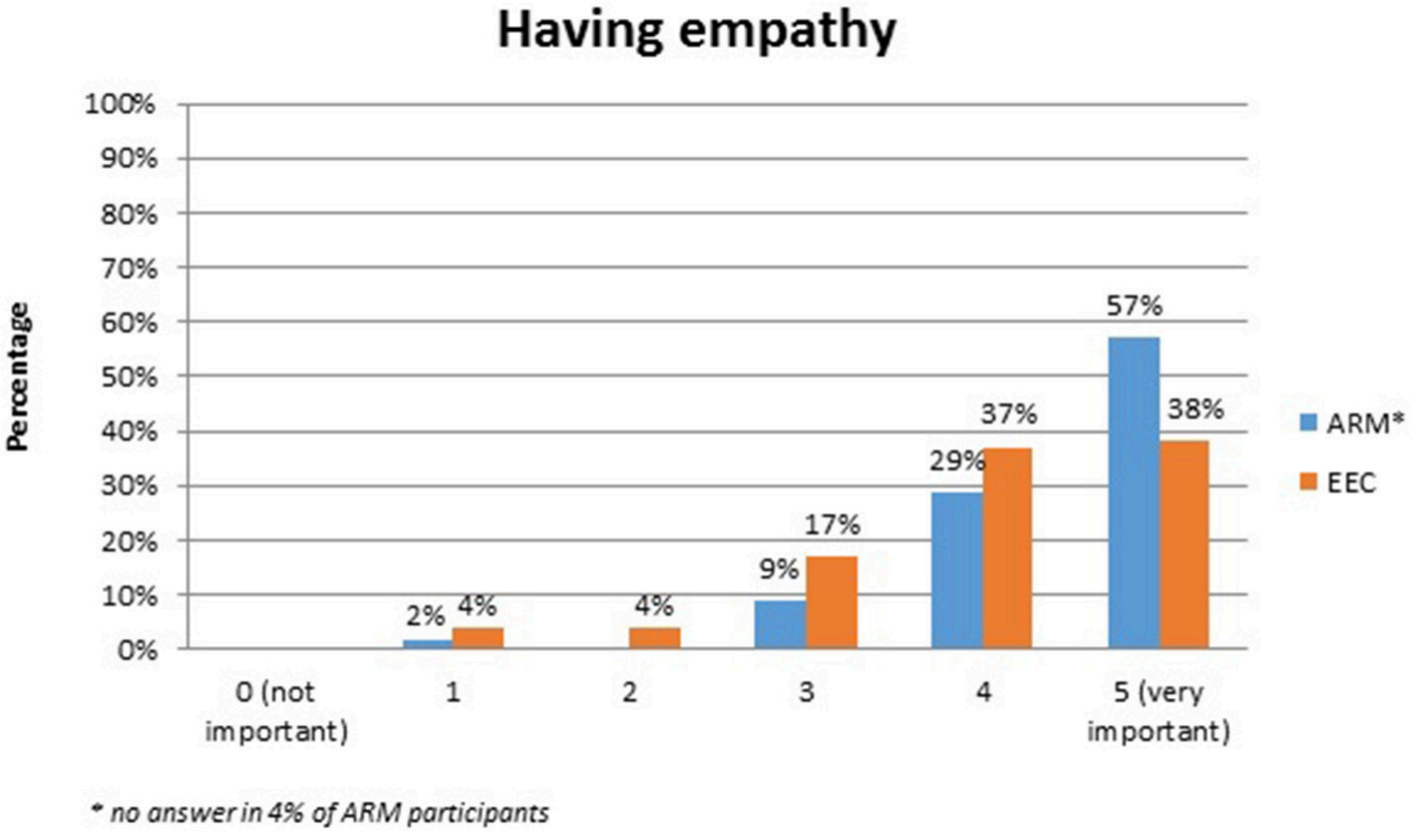
Expectations of adults with ARM and EEC regarding the soft skills of the treating physician: having empathy.

**Figure 5:**
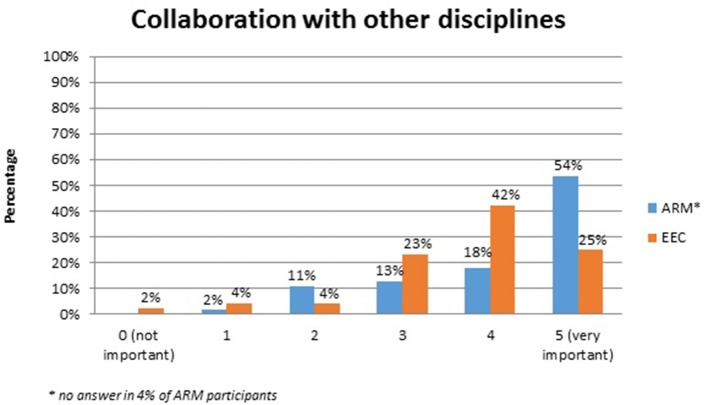
Expectations of adults with ARM and EEC regarding the soft skills of the treating physician: collaborations with other disciplines.

**Figure 6:**
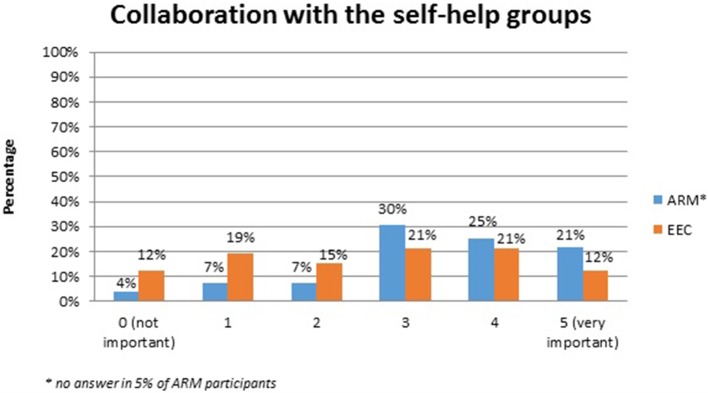
Expectations of adults with ARM and EEC regarding the soft skills of the treating physician: collaborations with the self-help groups.

**Figure 7:**
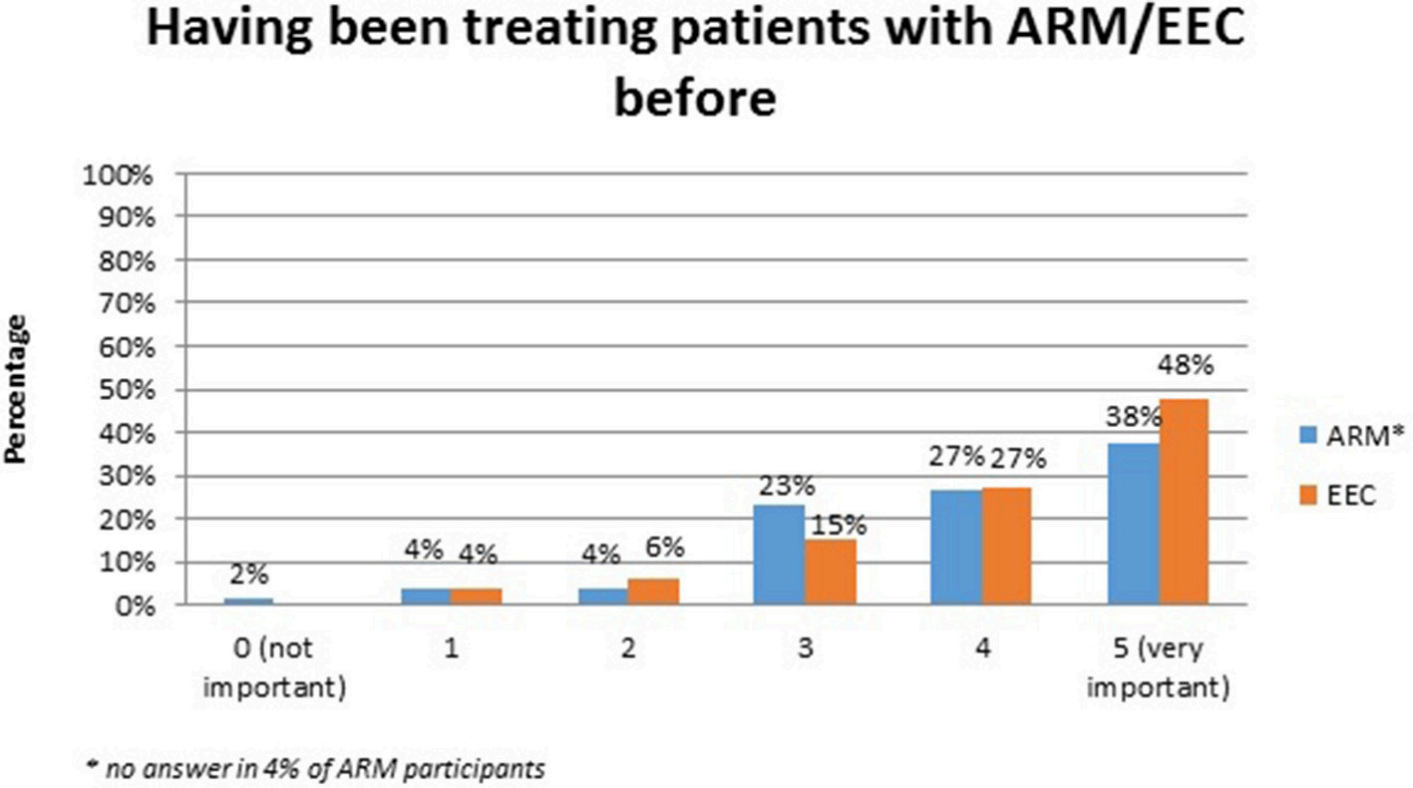
Expectations of adults with ARM and EEC regarding the soft skills of the treating physician: previous experience in treating ARM/EEC patients.

**Figure 8:**
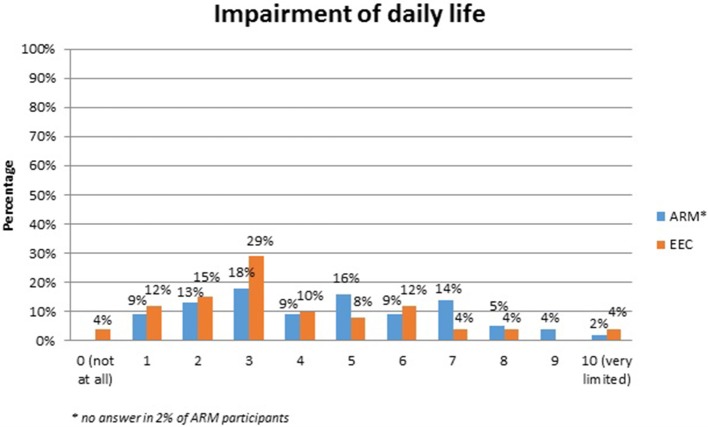
Impairment of daily life in adults with ARM and EEC.

No systematic difference was observed between the two anomaly groups regarding important topics for the participants themselves (Table 4). Fourty-five percent of the adults with ARM and 50% of the adults with EEC expressed their wish for counseling about the possibility and management of having own children. Continence consultations were warranted by 20 (36%) ARM and 13 (25%) EEC adults. Consultations regarding auxiliary medical supplies needed 19 (34%) ARM and 17 (33%) EEC individuals. Decisions for further reconstructive surgery was important for 12 (21%) ARM and 18 (35%) EEC adults. Counseling for a handicapped ID and public financial support was marked by 38% of ARM and 33% of EEC participants. Expectations regarding soft skills of the treating physician were marked as well (Figures 1–7). Main focus (very important = 5 and important = 4) was put on the item “professional knowledge” in over 90% of all participants [54 (96%) ARM/47 (90%) EEC] (Figure 1). Taking enough time [53 (95%) ARM/50 (96%) EEC] (Figure 2), paying attention to patients' concerns [52 (93%) ARM/50 (96%) EEC] (Figure 3), having empathy [48 (88%) ARM/39 (75%) EEC] (Figure 4) were very important for the participants. The qualities “collaboration with other disciplines or the self-help groups” (Figures 5, 6) as well as “having been treating patients with ARM/EEC before” (Figure 7) were judged more individually with a wider range of significance. Regarding impairment of daily life only 2% of ARM and 4% of EEC adults marked to be severely impaired during daily life. Figure 8 reproducibly shows that the particular values did not significantly differ between the both groups (*p* = 0.38). Twenty-six participants with ARM and 20 participants with EEC provided more detailed individual inside informations by using the free-text section. Self-reporting free-text information excerpts of ARM and EEC patients' personal experience with their medical care can be found translated in Data Sheet 3.

**Table 4:** Medical topics important for the participants (multiple answers allowed).

**Topic**	**ARM (*n* = 56)**	**EEC (*n* = 52)**	***P*-value**
			*p* = 0.6
Wish to have own children	25 (45%)	26 (50%)	
Questions regarding urological/nephrological issues	15 (27%)	14 (27%)	
Continence consultation	20 (36%)	13 (25%)	
Decisions for further reconstructive surgery	12 (21%)	18 (35%)	
Counseling for handicapped ID/public financial support	21 (38%)	17 (33%)	
Consultation regarding auxiliary medical supplies Other	19 (34%)	12 (23%)	
	3 (5%)	1 (2%)	

## Discussion

Current knowledge about the needs and concerns of adults with ARM and EEC regarding medical aid is still sparse. There are some current reports about transition programs for individuals with spina bifida in literature (11). As 40% of patients with urological conditions transit into adult medicine during a time period of 5 years, young adult patients with complex conditions such as myelomeningocele, traumatic brain or spinal cord injury and cerebral palsy seem to keep under pediatric urologists' supervision and care (16). The reasons for that observation remain unclear. However, from etiology and comorbidity of the conditions we may subsume that some individuals feel benefit from a more individual patient centered care, rather than from an organ centered one. Sixty-seven percent of participating EEC individuals stated that they had contacted an urologist. About 45% remain with their initially treating physician, mostly a pediatric urologist in a university center. Most of these contacts are approved as longstanding and faithful professional relationships (Data Sheet 3). From the survey's free text informations this fact can be proven in nearly all EEC patients' answers available (Data Sheet 3). From physicians' point there were some reports on congresses about the issues from adults with EEC deduced from transition program (17, 18). A particular problem for individuals with ARM is that in most pediatric surgery centers and children's hospitals in Germany there is an age limit of 16 or 18 years beyond which patients can no longer be taken care of. Most pediatric surgery centers are structured to offer medical and paramedical services to individuals born with ARM, services seldom available in general surgery departments. In the free-text opinions many ARM adults were dissatisfied for not having access to surgeons with knowledge of their disease and with adequate paramedical facilities to address their needs (Data Sheet 3). It is well-known that ARM individuals need urological care either due to primary urological comorbidity such as hypospadias or neurogenic bladder or due to secondary urological complications. In this study, 40% of the ARM participants stated that urological consultations were needed, however, this ratio might be even higher in other cohorts.

Using this German-wide online survey adult self-help group members with ARM and EEC were contacted by their corresponding self-help groups. A considerable amount of members could be motivated to take part in this self-administered questionnaire. The response rate of adults with EEC was significantly higher than for adults with ARM (*p* < 0.0001). EEC adults did recently have significantly less contact to their corresponding self-help group (*p* = 0.03), assumable more or less inactive members in the membership roster. Furthermore, the response rate of EEC participants (75%) was much higher compared to previous multicentre German-wide surveys in EEC issues (19, 20). Although presumably, the here used online questionnaire might have been more suitable and easier to complete for the contacted individuals. The addressed topics were generally accepted and probably more innocuous than the previously addressed intimate genital aspects. Epidemiological data such as age were comparable in both groups. The phenotype distribution and male-to-female ratio were comparable to literature for both anomalies (14, 15). Regarding contacts to medical subspecialities there were no significant differences for all listed physicians and paramedics (Tables 2, 3). However, in EEC either an adult (*n* = 35, 67%) or a pediatric (*n* = 22, 43%) urologists were contacted in a considerable percentage. Due to methodology of the questionnaire and anonymity of the data, overlapping results can unfortunately not be excluded. Forty-five percent of the ARM adults had also contact to urologists. Although in most cases medical contacts were judged to be helpful, a subgroup of females with ARM was not satisfied with gynecological consultation results. These were mainly females with non-isolated ARM and one individual with ARM and Hirschsprung diesase combined with additional anomalies. For this specific subgroup and in respect of the well-known complex genital situation a gynecological specialist with specific knowledge of ARM might be necessary to adequately address these patients' needs. Participants in both cohorts expressed to the same extent the need for consultations concerning the wish for own children, continence issues and further reconstructive surgery. In addition, questions regarding urological and nephrological issues, counseling for handicapped ID/public welfare financial support and help with auxiliary devices were important too.

Soft skills of medical professionals in surgical literature are rarely under debate (21). The expectations regarding patient-physician interactions in this study clearly show that beside expert knowledge physicians' soft skills are very important. Collaboration with other disciplines and experience with treating individuals with rare conditions before, however, seem not to be as important as suspected by physicians. The general question about the extent of daily life impairment, showed no significant difference between the ARM and EEC cohort. In contrast, in recent years, sophisticated Quality of life (QoL) investigations using validated questionnaires universely report a quasi-normal QoL of affected individuals with EEC and ARM (20, 22, 23). However, urinary incontinence, insufficient penile function and appearance and long-term complications seem to negatively impact QoL in all EEC individuals (20, 22). In ARM fecal incontinence seem to negatively influences physical and mental health-related QoL as well as self-efficacy (23). However, these psychological long-term issues were beyond the current analysis.

### Strength and Limitations

Strengths of this study are the nationwide and treating physician-independent data acquisition and the considerable and comparable sample sizes of both cohorts. Patient data were self-reported and thus an objective comparison with clinical findings or patient charts with the help of the treating physician was not intended. This methodology may generate the risk for data inaccuracy. However, as there is no ARM or EEC register in Germany including all affected individuals living in this country, a selection bias can not be ruled out. Furthermore, self-help group members are generally good informed, so the results relating to medical or social long-term issues must be treated with caution, as they migth not picture the general situation of all individulas with ARM and EEC living in Germany. Especially the response rate of the adult members of the German EEC self-help group was estonishingly high. In addition, not all participants answered the questionnaire completely and were therefore excluded from this study. Although the questionnaire as a whole was not validated, the pre-test performed has led to a reasonable face-validity. Finally, this pilot study provided many interesting information about the actual situation of ARM and EEC adults living in Germany and might therefore with its innovative explorative approach serve as the basis for further detailed studies.

## Conclusion

Participating members of self-help organizations seem to cope quite well with their congenital rare anomaly. About one third of all participating ARM and EEC adults needed psychological support within the last 2 years. Most contacts to the diverse treating physicians and paramedics seem to be helpful or at least neutral. Only a small minority stated that these contacts were not helpful at all. From the free-text answers we learned that adults with ARM in Germany seldom find expert surgeons leading to a general dissatisfaction with the medical aftercare in adulthood. Most adults with EEC, however, perceive longstanding care from their urological experts, mainly pediatric urologists, and therefore gain valuable continuous follow-up. The most prominent topic in both cohorts was any counseling about family planning and having own children. Participants stated the wish for expert consultation regarding reconstructive procedures, continence management, urological care and social welfare issues. Furthermore, expert consultations seem to be required specific for gynecological issues in females with non-isolated ARM or ARM with Hirschsprung disease and additional associated anomalies. Actual needs of adults with rare conditions must be better clarified to improve medical care beyond childhood and adolescence and to adequately optimize the existing medical care structures.

## Author Contributions

LB, NS, and EJ: Design of the questionnaire; NZ and AE: Data interpretation; AE, NZ, HR, BL, and JH: Writing the manuscript. Approval of the manuscript all authors.

### Conflict of Interest Statement

The authors declare that the research was conducted in the absence of any commercial or financial relationships that could be construed as a potential conflict of interest.
