# Supplementary material for: Needs Assessment in Care of Adults With Anorectal Malformations and Exstrophy-Epispadias Complex in Germany
Source: Front Pediatr. 2018 Dec 19;6:392. doi: 10.3389/fped.2018.00392 (PMC6306024; doi:10.3389/fped.2018.00392)
Supplement: Supplementary file 2 [file Data_Sheet_2.PDF]

Status of the follow-up for adults with anorectal malformations and Hirschsprung disease

Dear participant,

the goal of this short questionnaire is to obtain more information about the present situation of the medical care of adults with anorectal malformations and Hirschsprung disease in Germany. We wish to know if there are specific needs for the follow-up care and if so, in which areas. How satisfied are you and what are your wishes for your future medical care?

We are trying to put together a few important questions. We want to share the answers with specialists and decision makers responsible for health politics.

Of course, the participants will remain anonymous and we cannot identify who has already answered and who has not. For this reason we ask you to participate in this project only once.

If you would like to be informed of the results or wish to participate in future research projects, you may enter your e-mail address. This will be used only as contact information and it will be kept separate of the other data.

We thank you for participating
SoMA e.V. (Selbsthilfeorganisation für Menschen mit Anorektal-Fehlbildungen, http://www.soma-ev.de/)

The questionnaire contains 12 questions.

Demographics

[]Please state your gender:

Please chose only one answer:

- ○ Female
- ○ Male
- ○ Other (DSD = disorders of sex development)

[]Please state your age (in years):

Please enter only numbers.
Please give your age in the following box:

[ ]

[]Please state the main malformation:

Please chose only one answer:

- ◯ Anorectal malformation without other congenital malformations
- ◯ Anorectal malformation with at least one other malformation, for example heart, kidneys, spinal column, extremities (please explain in the comments section)
- ◯ Hirschsprung disease without other malformations
- ◯ Hirschsprung disease with at least one other malformation, for example heart, kidneys, spinal column, extremities (please explain in the comments section)

Provider

[] Please state if you have seen one of the mentioned health care provider below in the last 24 months (do not include consultations during meetings of self-help groups, only those e.g. in a clinic or office). Please state how satisfied you were with the visit.

Please select the corresponding answer for each specialty.

	No contact in the last 24 months	The consultation was helpful	The consultation was neutral	The consultation was NOT helpful
Pediatric Surgeon	○	○	○	○
General Surgeon	○	○	○	○
Proctologist	○	○	○	○
Urologist	○	○	○	○
Gynecologist	○	○	○	○
Continence counselor	○	○	○	○
Dietician	○	○	○	○
Physiotherapist	○	○	○	○
Psychotherapist/Psychologist	○	○	○	○
Nephrologist	○	○	○	○
Gastroenterologist	○	○	○	○
Osteopath	○	○	○	○
Family Doctor	○	○	○	○
Nurse practitioner	○	○	○	○
Social worker	○	○	○	○
Self-help group	○	○	○	○

[]

In the box below you may enter details about your degree of satisfaction (or dissatisfaction).

For example we are interested in: Why did you have the consultation? Why are you satisfied or dissatisfied? Why have you changed health care provider?

Please give your answers in the box below:

[] I would like to have medical/ therapeutic assistance or counseling in the following areas (you may select more than one area; you may a comments in the corresponding box).

Please comment when you choose an area.

- [ ] Would like to have a child
- [ ] Continence counselling
- [ ] Urology or nephrology questions
- [ ] Psychological help
- [ ] Decisions about surgery
- [ ] Financial counselling or disability assistance
- [ ] Counselling

Oher:

[] For me it is important that the caregiver…

Please select the corresponding answer:

	0 (not important)	1	2	3	4	5 (very important)
...is competent	○	○	○	○	○	○
...allows enough time for the consultation	○	○	○	○	○	○
...listens attentively	○	○	○	○	○	○
…has compassion	○	○	○	○	○	○
…works well with other specialists	○	○	○	○	○	○
...works with the self-help groups	○	○	○	○	○	○
...has worked with other patients with anorectal malformations/Hirschsprung disease	○	○	○	○	○	○

[] Please state what you always wanted to say regarding medical help for adults with anorectal malformations/ Hirschsprung disease:

Please write your comments in the box below:

[] Because of the anorectal malformations/Hirschsprung disease restricted in my quality of life:

0 (not a all) ○ ○ ○ ○ ○ ○ ○ ○ ○ ○ ○ 10 (very)

[] Please tell us in what areas you feel particularly restricted.

Please give your answers in the box below:

Self-help

[ ] Do you belong to a self-help group?

Please choose one answer

- Yes
- No

[] Please explain why you are a self-help group member or you are not.

Please give your answers in the box below:

Many thanks for your participation!
