# Supplementary material for: Needs Assessment in Care of Adults With Anorectal Malformations and Exstrophy-Epispadias Complex in Germany
Source: Front Pediatr. 2018 Dec 19;6:392. doi: 10.3389/fped.2018.00392 (PMC6306024; doi:10.3389/fped.2018.00392)
Supplement: Supplementary file 3 [file Data_Sheet_3.PDF]

Supplement 2: Self-reporting free-text information excerpts of patients´ personal experience with their medical care–(original in German, translated)

**Free-text information EEC**	**Free-text information ARM**
- Dissatisfaction: The specialist has no time for complex cases	- The follow-up in adolescence was sporadic, counseling non-existent. Medical treatment did not reveal anything special
- When I have problems or questions I consult my urologist who is familiar with the disease	- Towards the general surgeon I feel neutral. With the psychologist I am satisfied. I feel neutral towards the self-help group.
- The knowledge of adult urologists about bladder exstrophy is deficient. A well-regulated transition as is done with the cardiologists would be worthwhile.	- It is difficult to find a competent urologist.
- Most of our doctors are pediatric specialists so we are admitted to pediatric stations.	- As long as I was a child I had a good doctor as contact person. Now, as adult I have none :-(
- In general it must be said that doctors do not take the time to think and understand the problems and that standard protocols are inadequate. One must really be afraid of many doctors who overestimate their competence. For example I hope that in case of an accident no one has to open my abdomen or insert a catheter. Also many physicians are very insensitive and do not understand that they are dealing with traumatized people.	- Problem (dissatisfied): There is no good care for affected adults!! No one specializes in the problem! For this reason I stick with the pediatric surgeon and pediatric urologist. Unfortunately these specialists do not feel responsible when one is older than 18 or 21 years... There are also no good counselors in the area gynecology who have the knowledge or experience!
- I am very satisfied with my pediatric urologist since he or his substitute are always available and are also helpful over the phone.	- Most doctors have no idea from the diseases and have problems managing them
- The medical & therapeutical help is frequently only for children. As adults, the search for help is difficult and one needs luck to get information or a doctor.	- I have not yet found someone who can help ME. Everybody was very interested in hearing my medical history. I haven´t yet found a doctor who was competent for the follow-up. PITY!!!
- My urologist is very empathetic and cares intensely for my complains	- The self-help group is outstanding. It gives me self-assurance!!!
- I have had the same urologist for 20 years and I am very satisfied.	- A better network, better transition, contact addresses for the affected individuals and mostly centers of competence where e.g. surgeons, urologists and gynecologists work together. .
- There are very few experts.	- All must be in on hand! Build competence centers!
